# Phenotypic Effects of *FGF4* Retrogenes on Intervertebral Disc Disease in Dogs

**DOI:** 10.3390/genes10060435

**Published:** 2019-06-07

**Authors:** Kevin Batcher, Peter Dickinson, Michelle Giuffrida, Beverly Sturges, Karen Vernau, Marguerite Knipe, Sheida Hadji Rasouliha, Cord Drögemüller, Tosso Leeb, Kimberly Maciejczyk, Christopher A. Jenkins, Cathryn Mellersh, Danika Bannasch

**Affiliations:** 1 Department of Population Health and Reproduction, University of California-Davis, Davis, CA 95616, USA; klbatcher@ucdavis.edu (K.B.); kmaciejczyk@ucdavis.edu (K.M.); 2 Department of Surgical and Radiological Sciences, University of California-Davis, Davis, CA 95616, USA; pjdickinson@ucdavis.edu (P.D.); magiuffrida@ucdavis.edu (M.G.); bksturges@ucdavis.edu (B.S.); kmvernau@ucdavis.edu (K.V.); mfknipe@ucdavis.edu (M.K.); 3 Institute of Genetics, University of Bern, 3001 Bern, Switzerland; sheida.hadjirasouliha44@gmail.com (S.H.R.); cord.droegemueller@vetsuisse.unibe.ch (C.D.); tosso.leeb@vetsuisse.unibe.ch (T.L.); 4 Kennel Club Genetics Centre, Animal Health Trust, Kentford, Newmarket, Suffolk CB8 7UU, UK; chris.jenkins@aht.org.uk (C.A.J.); cathryn.mellersh@aht.org.uk (C.M.)

**Keywords:** inherited, intervertebral disc disease, breed, *Canis lupus familiaris*

## Abstract

Two *FGF4* retrogenes on chromosomes 12 (12-*FGF4*RG) and 18 (18-*FGF4*RG) contribute to short-limbed phenotypes in dogs. 12-*FGF4*RG has also been associated with intervertebral disc disease (IVDD). Both of these retrogenes were found to be widespread among dog breeds with allele frequencies ranging from 0.02 to 1; however, their additive contribution to disease is unknown. Surgical cases of IVDD (*n* = 569) were evaluated for age of onset, disc calcification, and genotypes for the *FGF4* retrogenes. Multivariable linear regression analysis identified the presence of one or two copies of 12-*FGF4*RG associated with significantly younger age at first surgery in a dominant manner. 18-*FGF4*RG had only a minor effect in dogs with one copy. Multivariable logistic regression showed that 12-*FGF4*RG had an additive effect on radiographic disc calcification, while 18-*FGF4*RG had no effect. Multivariable logistic regression using mixed breed cases and controls identified only 12-*FGF4*RG as highly associated with disc herniation in a dominant manner (Odds Ratio, OR, 18.42, 95% Confidence Interval (CI) 7.44 to 50.26; *p* < 0.001). The relative risk for disc surgery associated with 12-*FGF4*RG varied from 5.5 to 15.1 within segregating breeds and mixed breeds. The *FGF4* retrogene on CFA12 acts in a dominant manner to decrease the age of onset and increase the overall risk of disc disease in dogs. Other modifiers of risk may be present within certain breeds, including the *FGF4* retrogene on CFA18.

## 1. Introduction

The domestic dog exhibits a profound degree of phenotypic diversity in size. Two particular conditions affecting size, referred to as chondrodystrophy and chondrodysplasia, are characterized by shortened limbs and are common across many dog breeds [1,2]. The causes for both chondrodystrophy and chondrodysplasia were identified as two separate fibroblast growth factor 4 (*FGF4*) retrogenes on chromosome 12 (12-*FGF4*RG) and chromosome 18 (18-*FGF4*RG), respectively [1,2]. The most severe form of disproportionate dwarfism is seen in breeds that carry both *FGF4* retrogenes, such as Dachshunds, Basset Hounds, and Corgis [1,3]. FGF signaling is involved in early embryonal development [4,5], and appropriate levels of *FGF4* are necessary for normal limb formation [6]. Higher levels of *FGF4* transcripts were seen in dogs with either of the 12-*FGF4*RG or 18-*FGF4*RG insertions, leading to the conclusions that the *FGF4* retrogenes are expressed and that the short-limb phenotype is associated with overexpression during development [1,2]. Similarly in humans, achondroplasia, the most common form of dwarfism, is caused by the gain of function variants in the fibroblast growth factor receptor 3 that results in increased signaling [7,8].

In addition to shortened limbs, chondrodystrophic breeds are also characterized by chondroid metaplasia of the nucleus pulposus leading to premature degeneration and calcification of the intervertebral discs [9,10,11]. This degeneration predisposes chondrodystrophic dogs to intervertebral disc disease (IVDD), a debilitating disorder associated with protrusion or extrusion of intervertebral disc components into the vertebral canal, resulting in pain and/or neurological dysfunction [12]. Disc calcification may be visualized radiographically, and chondrodystrophic dogs with increased numbers of radiographically visible calcified discs have been shown to be at higher risk for clinical IVDD [13,14,15]. Calcification of the nucleus pulposus has also been described in older, non-chondrodystrophic dogs in the late stages of degeneration; however, this process generally occurs at an earlier age in the chondrodystrophic dog breeds [10,16,17].

While all breeds can be affected by IVDD, chondrodystrophic breeds are at particularly high risk [9,10,12]. Hansen classified the IVDD that occurs in the chondrodystrophic dog breeds as Type I, typified by acute extrusion of degenerate, often calcified nucleus pulposus through degenerate annulus fibrosis into the vertebral canal [10,18]. Hansen Type II IVDD generally occurs at a later age in larger breed (non-chondrodystrophic) dogs and more commonly involves chronic protrusion of degenerative disc material. Historically, type II IVDD has been reported to involve fibrous rather than chondroid disc degeneration. However, despite some specific differences in macro and microscopic pathology and in disease progression, Hansen’s original work and more recent studies have shown that chondroid metaplasia is a common underlying pathological process in both chondrodystrophic and non-chondrodystrophic breeds [9,19].

Current consensus supports the use of decompressive surgery to remove the disc material impinging on the spinal cord in dogs severely affected by IVDD [12,17], although the cost of surgery can be prohibitive for many owners. Dogs susceptible to IVDD may also suffer multiple disc herniations at different locations throughout their lifetimes [20]. While chondrodystrophic dog breeds with 12-*FGF4*RG alone, such as the French Bulldog and Beagle, are at high risk for IVDD, the chondrodysplastic breeds with 18-*FGF4*RG alone, such as the Scottish Terrier and the West Highland White Terrier, are not considered at high risk [1,10]. However, in dogs with both *FGF4* retrogenes, the contribution of 18-*FGF4*RG to disc degeneration and thus IVDD is unknown. It is also unclear whether or not the *FGF4* retrogenes act in a completely dominant manner or whether any additive effect exists. Since many breeds are homozygous for the *FGF4* retrogenes, the determination of the relative risk for intervertebral disc herniation is challenging. Segregating breeds and mixed breed dogs provide an opportunity to evaluate the risk of herniation in the presence of the retrogenes.

A broad analysis of allele frequency across dog breeds was performed for both *FGF4* retrogenes, identifying breeds that segregate or are fixed for one or both retrogenes. A referral hospital DNA database was utilized to obtain information from dogs that had received decompressive surgery for IVDD, and prospective samples were collected for two years from surgical cases to obtain a large, across breed sample of 569 dogs that had IVDD defined by surgery. These samples were genotyped for both 12-*FGF4*RG and 18-*FGF4*RG. Breed, weight, sex, age at time of first surgery, and the presence of calcified discs at the time of surgery were used to determine the contribution of 12-*FGF4*RG and 18-*FGF4*RG to disease phenotype using linear and logistic regression. A separate logistic regression was performed in mixed breed dogs to determine characteristics contributing to IVDD surgery itself, and a relative risk for 12-*FGF4*RG was calculated in segregating breeds.

## 2. Materials and Methods

### 2.1. Samples

Unused blood samples were collected from the University of California (UC) Davis Veterinary Medical Teaching Hospital (VMTH) hematology laboratory from 5 November 1999 to 1 February 2016 irrespective of IVDD diagnosis in a random fashion and entered in a repository. After 1 February 2016, the Bannasch laboratory began actively soliciting blood samples from IVDD cases seen at the VMTH. For the purpose of this study, samples that were collected prior to 1 February 2016 were marked as retrospective, while samples collected after 1 February 2016 were marked as prospective. Medical records for all samples in the DNA repository were queried from the VMTH database for evidence of decompressive surgery, and all surgical cases were included in the study. Samples collected from the VMTH were obtained under UC Davis IACUC protocols 12693, 15356, 18561, and 20356.

Medical information retrieved for each case included breed, sex, date of birth, weight, date of procedure, surgical procedure performed, anatomical location of surgery, a summary of findings from any medical imaging performed (Myelography, Computed Tomography, CT, Magnetic resonance Imaging, MRI, or Radiography), and a description of disc material from the surgery report. All non-disc related cases were excluded based on findings indicated in the surgery reports. All breed information was owner reported when presenting at the VMTH. All Miniature and Toy Poodles were treated as one breed, and all Dachshund varieties were treated as one breed; however, based on weight, all but two Dachshunds were miniature in size. The total numbers of dogs from the affected breeds were counted from the DNA repository in order to determine the relative breed representation of the surgical cases in the repository.

Breed-specific allele frequencies for the *FGF4* retrogenes were determined using 2333 samples from the UC Davis DNA repository irrespective of IVDD diagnosis. Additional samples for breed-specific allele frequencies were acquired from the Vetsuisse Biobank, University of Bern DNA repository without health information. Additional samples for the Dachshund breed in the United Kingdom (UK) were genotyped from a collection from the Animal Health Trust.

### 2.2. Phenotyping and Genotyping

Cases were classified into one of three categories based on information obtained from medical imaging and surgery reports. If the disc material removed at the time of surgery was described as calcified, mineralized, chondroid, or in other such similar terms, the case was classified as ’Group A’. The case was also classified as Group A if there was evidence of intervertebral disc calcification on the radiograph or CT report. If the disc material was described as annular or fibrous and there was no evidence of intervertebral disc calcification in any imaging, or if the disc material was described as hydrated or liquid, the case was classified as ’Group B’. Finally, if there was insufficient information to make any determination as to the nature of the IVDD due to a missing or nondescript surgery report and/or no vertebral column radiographs or CT imaging available, the case was classified as ’unknown’. Cases were also separately categorized based on the presence of calcified discs visible on radiograph irrespective of surgery report. DNA was extracted from whole blood samples using a Gentra Puregene DNA extraction kit (Qiagen, Valencia, CA, USA). Genotyping for 12-*FGF4*RG and 18-*FGF4*RG was performed using a PCR based assay as previously described [1] or through commercially available genotyping at the UC Davis Veterinary Genetics Laboratory.

### 2.3. Statistical Analysis

Descriptive statistics including interquartile ranges, median and 95% Confidence intervals for weight and age at surgery were obtained using GraphPad Prism 7.03 for Windows (GraphPad Software, La Jolla, CA, www.graphpad.com). The Mann-Whitney U test was used to compare weights across genotype statuses and between Groups A and B. A Chi-Squared test was used to test for significant differences in allele frequencies between Groups A and B. These analyses were also performed using GraphPad Prism 7.03 for Windows.

Multivariable linear regression analysis was used to identify characteristics associated with age at time of surgery. In order to evaluate breed contribution, the three most frequent breed representatives were used and compared to et al. (Table 1). Age was the dependent variable, and sex, reproductive status (spayed or neutered vs intact), body weight, breed (French bulldog, Dachshund, mixed breed, other pure breed), 12-*FGF4*RG status (zero copies, one copy, two copies) and 18-*FGF4*RG status (zero copies, one copy, two copies) were independent variables. Reference categories for categorical variables were mixed breed for breed and zero copies for retrogene variables. Weight was centered at population mean body weight (13.0 kg). Difference column indicates the estimated difference in age at surgery for each additional 5 kg of body weight. Univariable analyses were performed first and all independent variables with Wald *p* < 0.2 were tested for inclusion in the multivariable model. A backward elimination approach to model building was used, with variables retained in the final model if *p* < 0.05, or if they were identified as confounding variables (defined as >15% difference in coefficients). Interactions between the main effects were tested. Results were reported as differences in mean ages at surgery and surrounding 95% confidence intervals (CI).

Multivariable logistic regression was used to identify characteristics associated with the presence of disc calcification as determined by the medical record. Breeds were defined as previously. The presence of at least one calcified disc was the dependent variable and sex, age at surgery, body weight, reproductive status, breed (French bulldog, Dachshund, mixed breed, other pure breed), 12-*FGF4*RG status (zero copies, one copy, two copies), and 18-*FGF4*RG status (zero copies, one copy, two copies) were independent variables. Reference categories for categorical variables were mixed breed for breed and zero copies for retrogene variables. Weight was centered at population mean body weight (13.0 kg), and the OR represents each additional 5 kg of body weight. Univariable analyses were performed first and all independent variables with Wald *p* < 0.2 were tested for inclusion in the multivariable model. Both retrogene variables were forced into the model and other variables were retained using a backward elimination, with variables retained in the final model if *p* < 0.05, or if they were identified as confounding variables (defined as >15% difference in coefficients). Interactions between the main effects were tested. Results were reported as odds ratios (OR) and surrounding 95% CIs. Regression analyses were performed using Stata statistical software (StataCorp. 2015. Stata Statistical Software: Release 14. College Station, TX, USA.

A separate logistic regression among mixed breed dogs to determine characteristics associated with surgery for IVDD was also performed. All mixed breed dogs that had been collected retrospectively were 10 years or older at the time of their last visit and were free of any IVDD diagnosis were used as controls, while retrospectively collected mixed breed dogs that had received decompressive surgery for IVDD were used as cases.

Breed-specific relative risks associated with 12-*FGF4*RG were calculated for breeds that segregated the retrogene (allele frequency <0.5 and >0.05) and had a large enough sample size (number of surgeries >4). Only cases and controls that were sampled retrospectively with respect to IVDD (prior to 1 February 2016) were included. Dogs that were at least 10-years-old with no diagnosis of IVDD as of their last hospital visit were used as controls. 12-*FGF4*RG was treated as a dominant allele for relative risk calculations, where cases and controls with one or two copies of 12-*FGF4*RG were treated equally. Relative risk calculations were conducted in GraphPad Prism.

## 3. Results

### 3.1. Allele Frequency

To identify breeds that segregate the *FGF4* retrogenes and determine allele frequencies, 3223 additional dogs from 75 different breeds were genotyped (Supplementary Table S1). 12-*FGF4*RG was identified in at least one dog from 40 different breeds, while 18-*FGF4*RG was found in 32 different breeds. At least one dog from 23 of the 75 breeds tested had both 12-*FGF4*RG and 18-*FGF4*RG.

### 3.2. Description and Genotype of Disc Decompressive Surgical Cases

In order to dissect *FGF4* retrogene contribution to disc herniation, both retrospective and prospective cases requiring disc decompressive surgery were evaluated. Six hundred and twelve unique cases were identified that had undergone spinal cord decompressive surgery and had DNA available for genotyping. Forty-three cases were identified as non-disc related (16 neoplasia, nine infectious or inflammatory, eight vertebral anomalies, four trauma, and six miscellaneous) and were excluded from further analysis. The final dataset contained 569 surgical cases, 272 of which were collected retrospectively (prior to 11 February 2016), and 297 that were collected prospectively (after 11 February 2016). Of the 569 cases, 56 (9.82%) had received two or more decompressive surgeries at the VMTH. The dataset included dogs from 61 different breeds, as well as 127 mixed breed dogs (Table 1). There were 257 (45.3%) neutered males, 227 (40.0%) spayed females, 68 (12.0%) intact males, and 16 (2.8%) intact females. The median age at time of surgery for all cases was 6.4 years (interquartile range, IQR, 4.6–8.8). The median body weight was 8.6 kg (IQR 6.1–14.0). Breed IVDD surgery prevalence among cases collected retrospectively is also presented in Table 1.

All cases were genotyped for 12-*FGF4*RG and 18-*FGF4*RG: 75.2% of the cases had either one or two copies of 12-*FGF4*RG (allele frequency 0.636), and 57.3% had either one or two copies of 18-*FGF4*RG (allele frequency 0.509). Segregation of 12-*FGF4*RG was found in most breeds where it was identified, although several had an extremely high allele frequency, specifically the Dachshund, Beagle, French Bulldog, Pembroke Welsh Corgi, Basset Hound, and Spaniel breeds (Table 1). Several breeds were identified without 12-*FGF4*RG, including Miniature Pinschers, Doberman Pinschers, Rottweilers, and Pomeranians.

**Figure 1:**
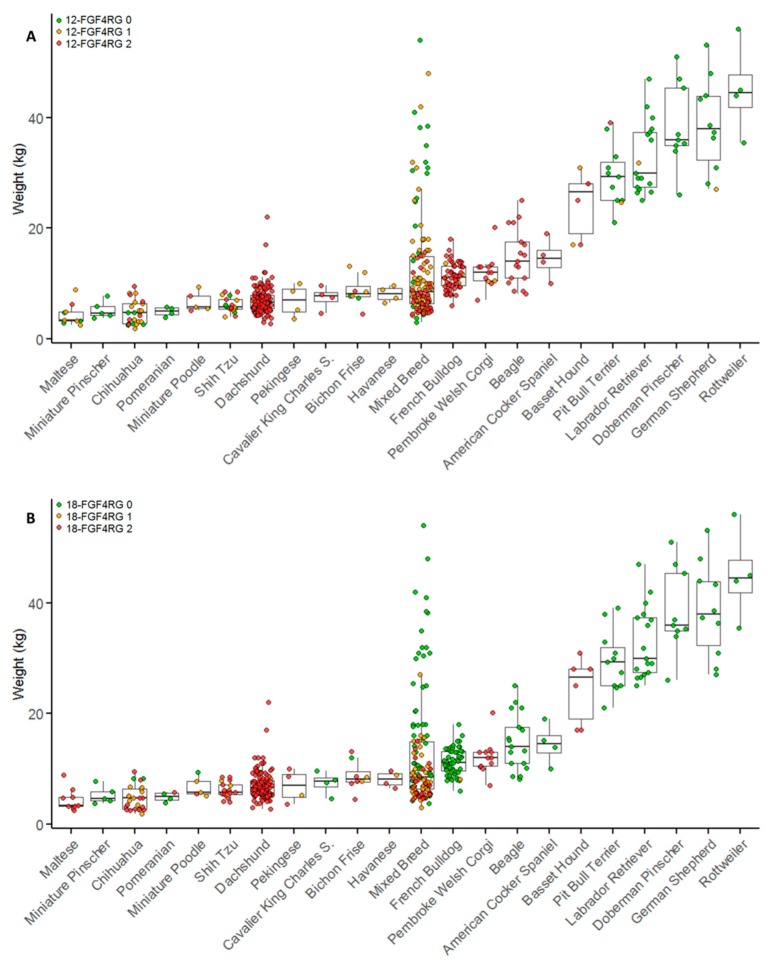
Breed and genotype distribution of surgical IVDD cases by body weight. Breeds with fewer than four cases are not included in this figure. Breeds are plotted in order of ascending median weights and colored by 12-*FGF4*RG genotype (**A**) and 18-*FGF4*RG genotype (**B**) *n* = 510). Red indicates two copies of each retrogene, orange indicates one copy, and green indicates zero copies.

The *FGF4* retrogenes have been implicated in leg length and height in dogs. Although height data were not collected for these animals, weight is routinely collected on all hospitalized cases and can be used to estimate overall size of the dog. Figure 1 shows the weight and genotype status of cases within each breed. Based on weight, there were 146 miniature Dachshunds and two standard Dachshunds included. A wide distribution of weight and genotype status was observed in the mixed breed dogs (Figure 1). Overall, dogs with one copy of 12-*FGF4*RG (median 8.6 kg, IQR 6.0–12.0) or two copies of 12-*FGF4*RG (median 7.8 kg, IQR 5.9–11.0) weighed significantly less than dogs with zero copies of 12-*FGF4*RG (median 25.4 kg, IQR 7.4–37.0; *p* < 0.001). There was no significant difference in weight between dogs with one or two copies of 12-*FGF4*RG (*p* = 0.143). Dogs with one copy of 18-FGF4RG (median 6.9 kg, IQR 5.5–8.8) or two copies of 18-*FGF4*RG (median 6.6 kg, IQR 5.4–8.6) weighed significantly less than dogs with zero copies of 18-*FGF4*RG (median 14.3 kg, IQR 10–30.0; *p* < 0.001). There was no significant difference in weight between dogs with one or two copies of 18-FGF4RG (*p* = 0.234).

This analysis was performed within the mixed breed dog population since purebreds have strong selection for specific sizes. Mixed breed dogs with one copy of 12-*FGF4*RG (median 9.1 kg, IQR 6.6–14.3) or two copies of 12-*FGF4*RG (median 7.1 kg, IQR 5.5–8.8) weighed significantly less than dogs with zero copies of 12-*FGF4*RG (median 22.6 kg, IQR 6.6–32.8; *p* < 0.001, *p* = 0.005). Mixed breed dogs with two copies of 12-*FGF4*RG also weighed significantly less than mixed breed dogs with only one copy (*p* = 0.001). Mixed breed dogs with one copy of 18-*FGF4*RG (median 7.6 kg, IQR 6.1–10) or two copies of 18-*FGF4*RG (median 7 kg, IQR 5.5–9.4) weighed significantly less than dogs with zero copies of 18-*FGF4*RG (median 16 kg, IQR 9–30.4; *p* < 0.001). There was no significant difference in weight between dogs with one or two copies of 18-*FGF4*RG (*p* = 0.159).

**Figure 2:**
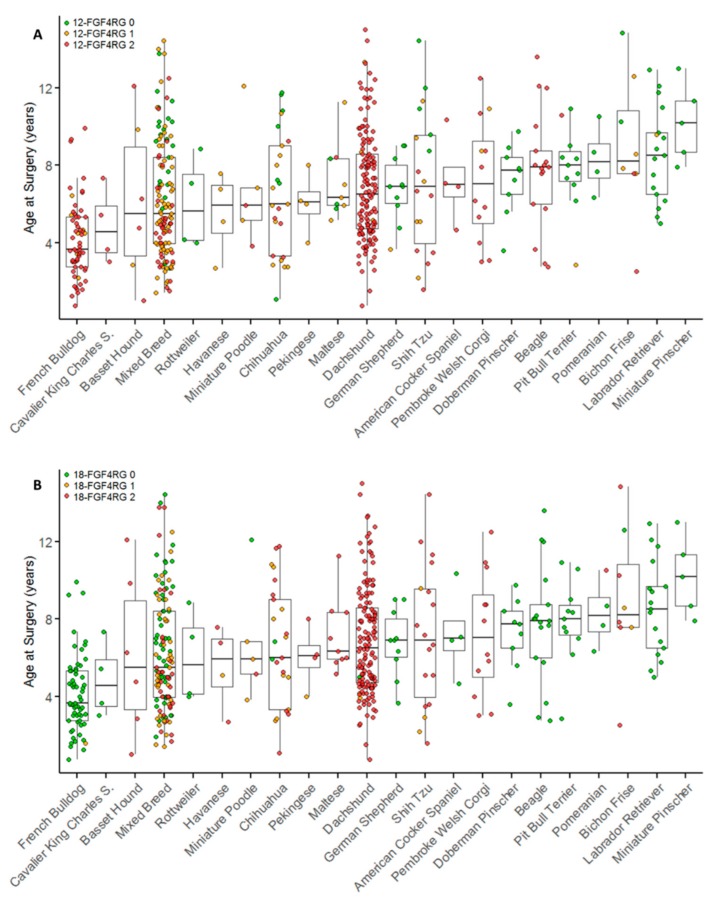
Breed and genotype distribution of surgical IVDD cases by age at surgery. Breeds are plotted in order of ascending median age at surgery. Individuals are colored by 12-*FGF4*RG genotype (**A**) and 18-*FGF4*RG genotype (**B**). Breeds with fewer than four cases are not included (*n* = 510). Red indicates two copies of each retrogene, orange indicates one copy, and green indicates zero copies.

Surgeries were primarily performed at the thoracolumbar region, with 413 (72.6%) surgeries performed between T9 and L6, 143 (25.1%) between C1 and T1 and 13 (2.3%) at the lumbosacral junction. Anatomical localization of surgical procedure as a percentage of all surgeries performed for each genotype category is shown in Supplementary Figure S1. The age at first surgery for breeds with more than four surgical cases compared with 12-*FGF4*RG and 18-*FGF4*RG genotypes is presented in Figure 2.

### 3.3. Linear Regression of Age at Surgery

Univariable and multivariable linear regression were performed to determine the effect of each retrogene on age at time of surgery. On multivariable linear regression, 12-*FGF4*RG status and breed category had the largest effect on age at surgery, followed by weight and 18-*FGF4*RG (Table 2). Dogs with one copy of 12-*FGF4*RG (Table 2; *p* < 0.001) or with two copies of 12-*FGF4*RG (Table 2; *p* < 0.001) were significantly younger at the time of surgery when compared to dogs with zero copies of 12-*FGF4*RG. There was no significant difference in age at surgery between dogs with one versus two copies of 12-*FGF4*RG (mean difference −4.6 months, 95% CI −12.7 to 3.5; *p* = 0.266). Adjusting for other variables, French Bulldogs were significantly younger at the time of surgery than mixed breeds (Table 2; *p* < 0.001), Dachshunds (mean difference −37.3 months, 95% CI −50.3 to −24.4; *p* < 0.001), and other pure breeds (mean difference −30.1 months, 95% CI −41.8 to −18.3; *p* < 0.001). Dachshunds were significantly older at surgery when compared to mixed breed dogs (Table 2; *p* = 0.014). Dogs with one copy of 18-*FGF4*RG (Table 2; *p* = 0.014) were significantly younger at the time of surgery when compared to dogs with zero copies of 18-*FGF4*RG. There was no significant difference in age at surgery between dogs with zero versus two copies of 18-*FGF4*RG (Table 2; *p* = 0.239) or between dogs with one versus two copies of 18-FGF4RG (mean difference 7.6 months, 95%CI −2.29 to 17.5; *p* = 0.132). 

### 3.4. Evaluation of Disc Calcification

Surgical cases were then categorized based on evidence of disc calcification into Group A and Group B. Table 3 shows genotyping results and characteristics for dogs based on group classification. Dogs in Group A (calcified disc material on radiograph or at surgery) were smaller (median 8.1 kg, IQR 6.1–12.8)(*p* < 0.001, effect size 14 kg, 95% CI 8.7 to 19.3) and younger (median 5.5 years, IQR 3.9–8.0)(*p* < 0.001, effect size 2.7 years, 95% CI 1.8 to 3.6) at the time of surgery compared to the Group B (non-calcified disc material) dogs (median 25 kg, IQR 8.5–38)(median 9 years, IQR 6.4–11). 12–*FGF4*RG was more common in Group A than in Group B; the allele frequency of 12-*FGF4*RG was 0.765 in Group A and 0.149 in Group B (χ² = 149.9, *p* < 0.001). 18-*FGF4*RG was also more common in Group A than in Group B with an allele frequency of 0.587 and 0.160 (χ² = 61.6, *p* < 0.001). Forty-six cases classified as Group A had zero copies of 12-*FGF4*RG. The breeds represented in this category included Labrador Retriever, Doberman Pinscher, German Shepherd, Pit Bull Terrier, Rottweiler, and Pomeranian, as well as several individual cases from other breeds and mixed breed dogs.

Within Group A, dogs with zero copies of 12-*FGF4*RG were significantly older at time of surgery than Group A dogs with one copy of 12-*FGF4*RG (mean difference 22.7 months, 95% CI 10.4 to 35.0; *p* < 0.001) or two copies of 12-*FGF4*RG (mean difference 20.2 months, 95% CI 9.5 to 30.9; *p* < 0.001). There was no significant difference in age at surgery between Group A dogs with one versus two copies of 12-*FGF4*RG (*p* = 0.372). One hundred and thirty Group A dogs had zero copies of 18-*FGF4*RG. There was no significant difference in age at time of surgery between Group A dogs with zero copies of 18-*FGF4*RG and Group A dogs with one copy of 18-*FGF4*RG (*p* = 0.0.240) or two copies of 18-*FGF4*RG (*p* = 0.202). However, Group A dogs with one copy 18-*FGF4*RG were significantly younger at time of surgery than group A dogs with two copies of 18-*FGF4*RG (mean difference 11.8 months, 95% CI −22.3 to −1.3; *p* = 0.026).

Radiographic screenings for calcified discs are used by some breeding programs in an attempt to reduce the incidence of IVDD [21]. Therefore, we compared the characteristics of cases with and without calcified discs defined by radiography. Four hundred and twenty-three of the surgical cases had radiographic reports available for review. Calcified discs were observed significantly (*p* < 0.001) more often in dogs with two copies of 12-*FGF4*RG (84.8%; 190/224) than in dogs with one copy (63.8%; 51/80) or zero copies (18.5%; 22/119) of 12-*FGF4*RG. Univariable and multivariable logistic regression was performed in order to determine what characteristics had an effect on disc calcification (Table 4). The main contributor to disc calcification was the presence of 12-*FGF4*RG. The odds of disc calcification increased with increasing number of copies of 12-*FGF4*RG. When compared to dogs with one copy of 12-*FGF4*RG, dogs with two copies had 2.5 times greater odds of disc calcification (OR 2.46, 95% CI 1.21 to 5.03; *p* = 0.013). Other significant contributors to disc calcification were age and breed. The rate of having at least one calcified disc differed significantly (*p* < 0.001) by breed. At least one calcified disc was observed in 105/116 (90.5%) Dachshunds, 36/51 (70.6%) French Bulldogs, 51/82 (60.2%) mixed breeds, and 71/174 (40.8%) other pure breeds. Dachshunds had significantly higher odds of disc calcification versus other pure breeds (OR 2.53, 95% CI 1.01 to 6.36; *p* = 0.048) and versus French bulldogs (OR 5.03, 95% CI 1.54 to 16.46; *p* = 0.007). While copy number of 18-*FGF4*RG was significant on univariable analysis, on multivariable analysis adjusting for other variables there was no significant difference in odds of disc calcification when comparing dogs with zero copies of 18-*FGF4*RG to dogs with one or two copies (Table 4), or when comparing dogs with one versus two copies (OR 1.56, 95% CI 0.58 to 4.20; *p* = 0.376).

### 3.5. Relative Risk

Since a large number of mixed breed dogs were available from the retrospective collection that had disc decompressive surgery (N = 46) and suitable controls were available (N = 460: mixed breed dogs aged 10 years or older without history of disc herniation), univariable and multivariable logistic regression was performed to determine which factors are associated with risk of decompressive surgery for IVDD. On univariable logistic regression, 12-*FGF4*RG, 18-*FGF4*RG, and weight were significantly associated with IVDD surgery (Table 5). However, under multivariable analysis, 12-*FGF4*RG status was the only contributor significantly associated with IVDD surgery. There was no significant difference in odds of IVDD surgery when comparing dogs with one or two copies of 12-*FGF4*RG (OR 2.40, 95% CI 0.89 to 6.46; *p* = 0.083).

Because 12-*FGF4*RG was found to be the main contributor to both age at IVDD surgery and disc calcification among surgical cases and was the only factor significantly associated with IVDD surgery in mixed breed dogs, a breed-specific relative risk associated with the presence of 12-*FGF4*RG was then calculated. The high allele frequency of 12-*FGF4*RG among chondrodystrophic breeds, such as Dachshunds and French Bulldogs, precluded the calculation of a relative risk, and, therefore, only breeds that segregated 12-*FGF4*RG (allele frequency <0.5) and mixed breed dogs were included (Table 6). Only samples that had been collected randomly with respect to IVDD were used for relative risk calculations. Mixed breeds with 12-*FGF4*RG had the highest associated relative risk for IVDD at 15.1.

## 4. Discussion

Both 12-*FGF4*RG and 18-*FGF4*RG are common across many dog breeds, as they were found in at least one dog in 53% and 43%, respectively, of the 75 breeds tested. Among the dogs treated surgically for IVDD, dogs that carried at least one copy of 12-*FGF4*RG were significantly younger, smaller and more likely to have radiographically calcified discs than the dogs without 12-*FGF4*RG. 12-*FGF4*RG was the only retrogene with a statistically significant effect on disc calcification in an additive manner. Age and breed also had modest effects on disc calcification. Under multivariable logistic regression, 12-*FGF4*RG was the only factor contributing to IVDD surgery in mixed breed dogs. The relative risk for 12-*FGF4*RG varied among segregating breeds, with mixed breed dogs carrying 12-*FGF4*RG the most at risk for IVDD.

The 12-*FGF4*RG has been described in association with the chondrodystrophic phenotype [1], as previously defined clinically and pathologically by Hansen, Braund, and others [10,11]. Consistent with these historical data, presence of the 12-*FGF4*RG in this large data set was found to be significantly associated with four major clinico-pathological phenotypes associated with chondrodystrophy-associated IVDD; specifically, small size, anatomical location of IVDD, early age of onset, and presence of calcification. IVDD in 12-*FGF4*RG carrying dogs was mostly located in the thoracolumbar region, while only 14.2% of the extruded discs localized to the cervical region. Hansen observed that only 15% of the extruded discs in the chondrodystrophic breeds were cervical, and other studies have also shown that thoracolumbar herniation is most common in the chondrodystrophic breeds [18,22,23]. In contrast, among the dogs with zero copies of 12-*FGF4*RG, 42.2% of the extruded discs localized to the cervical region. The caudal cervical region, in particular, was the most affected, although thoracolumbar and lumbosacral IVDD was also present among the dogs with zero copies of 12-*FGF4*RG, results which are consistent with previous studies in non-chondrodystrophic breeds [18,23,24].

While age at time of surgery across all breeds was significantly lower for 12-*FGF4*RG dogs, significant differences were present within specific chondrodystrophic breeds suggestive of additional factors, either genetic or environmental, that may contribute to overall disease presentation. As has been described previously, French Bulldogs had a mean age at time of surgery significantly lower than other breeds, at 4.1 years [25]. Alterations in WNT pathway signaling have been consistently implicated in aging and degeneration of the intervertebral disc [26,27,28]. Down regulation of WNT signaling has been described specifically in chondrodystrophic dog degenerate nucleus pulposus [27], and a downregulating frameshift variant in the WNT pathway gene *Dishevelled 2* (*DVL2*) was also recently identified and associated with screw tail and brachycephaly in Bulldogs, French Bulldogs, and Boston terriers [29]. Bulldogs and Boston Terriers rarely carry the 12-*FGF4*RG and are rarely reported with IVDD; however, it is interesting to speculate that the significantly earlier onset of IVDD in French Bulldogs carrying the 12-*FGF4*RG may be related to additional perturbation of WNT signaling exacerbating *FGF4* retrogene-related pathology.

Dachshunds, the breed with the highest prevalence of disc disease in this study and elsewhere, surprisingly had a significantly older age of onset than mixed breeds. While fewer individuals were identified from other breeds, there is a trend that breeds with a high allele frequency of 12-*FGF4*RG have a later median age of onset of disc herniation. It is possible that breeds with high allele frequencies of 12-*FGF4*RG, such as Beagles and Dachshunds, have undergone additional selection with younger onset affected animals being more likely to be excluded from the breeding pool. It is also possible that owners treat their dogs differently since they are aware of the risk of disc herniation within these breeds. Within breed selection for protective effects could also explain why mixed breed dogs suffer the greatest relative risk for IVDD associated with 12-*FGF4*RG, as they would not benefit from any protective alleles.

The original classification of type I and type II IVDD made by Hansen was done based on histopathological examinations of intervertebral discs and signalment. For this study, in the absence of histopathology, when descriptions of calcified or mineralized disc material were available from the surgery or radiographic reports, the cases were classified as Group A or Group B. Under this classification system, the majority (87%) of dogs in Group A had at least one copy of 12-*FGF4*RG, consistent with a dominant mode of inheritance. Interestingly, 46 out of the 378 cases classified as Group A had no copies of 12-*FGF4*RG. Previous studies have shown that Hansen type I IVDD can occur in non-chondrodystrophic breeds, and, more recently, it has also been shown that the histopathological progression of disc degeneration is similar between the chondrodystrophic and non-chondrodystrophic breeds [17,19,22]. Therefore, it is reasonable to expect that some older non-chondrodystrophic dogs could present with clinical IVDD resembling that seen in the chondrodystrophic breeds. In this study, the Group A dogs with zero copies of 12-*FGF4*RG were on average 22 months older than the Group A dogs with one or two copies of 12-*FGF4*RG. It is unclear whether non-chondrodystrophic dogs presenting with either calcified intervertebral discs or chronic disc protrusions are reflections of a spectrum of presentations within a common phenotype, similar to heterogeneity that may be seen even within chondrodystrophic breeds, or whether additional genetic factors may be present in non-chondrodystrophic dogs, resulting in histopathologically similar end points of disc degeneration with a later onset of disease.

Although the current and previous data support 12-*FGF4*RG as the chondrodystrophy locus, many chondrodystrophic breeds expressing the 12-*FGF4*RG also carry the 18-*FGF4*RG, possibly reflecting prolonged breeding selection for a short limbed phenotype through two different loci (Supplementary Table S1; Table 1). The effect of 18-*FGF4*RG was modest and only significant in one copy and not two. The number of dogs in this category (heterozygous for 18-*FGF4*RG) was low (N = 73) compared to the number of homozygous animals (zero copies 18-*FGF4*RG N = 243; two copies 18-*FGF4*RG N = 253). These differences in allele frequency are reflective of the allele frequency results within breeds reported here and elsewhere [2]. The fact that two copies of 18-*FGF4*RG does not significantly reduce the age of onset of IVDD argues that the significant effect seen in this dataset for one copy is less likely to be biologically significant. Logistic regression using calcification as an outcome also found that the addition of 18-*FGF4*RG did not improve the regression equation compared to 12-*FGF4*RG alone. Multivariable logistic regression also showed that 18-*FGF4*RG did not significantly contribute to IVDD surgery in mixed breed dogs, where 12-*FGF4*RG alone explained the outcome. While this study does not rule out that 18-*FGF4*RG is contributing to the IVDD disease phenotype in minor ways, such as a younger age of onset (13 months) in dogs with one copy of 18-*FGF4*RG, the effect of 12-*FGF4*RG was found to be far greater on all aspects of the disease.

Progression of the nucleus pulposus from normal to a radiographically visible degenerate and mineralized pathology appears to be under the influence of copy number of 12-*FGF4*RG since there is an additive effect. This is in contrast to the effect on age of onset of herniation, which is the same between dogs with one or two copies of 12-*FGF4*RG. In previous work evaluating height in the Nova Scotia Duck Tolling Retriever, the effect of 12-*FGF4*RG was also additive. Beagle neonatal puppies with two copies of 12-*FGF4*RG were previously shown to have 20-fold higher expression of *FGF4* in the intervertebral disc compared to Cane Corso neonatal puppies with no *FGF4* retrogenes [1]. It is possible that continued high expression in the disc in adults is contributing to the rate of mineralization in an additive fashion but that the presence of degeneration is enough to predispose dogs to herniation.

The mechanisms underlying the differential phenotypes associated with the *FGF4* retrogenes remain to be elucidated. The relatively minimal effects of 18-*FGF4*RG on disc disease compared to that of 12-*FGF4*RG could be due to differences in expression patterns between the two *FGF4* retrogenes. Although retrogenes are often regarded as non-expressing pseudogenes due to the frequent lack of defined regulatory elements [30], the 5′ end of the *FGF4* retrogenes contains a highly conserved CpG island which is predicted to function as a promotor [31,32]. Both *FGF4* retrogenes have been shown to be transcriptionally active, although associated clinical phenotypes appear to be different [1,2]. It was previously theorized that expression of 12-*FGF4*RG in the intervertebral disc is based on the chromosomal environment in which it was inserted, since all nearby genes were shown to be expressed in the intervertebral disc [1]. Temporal and tissue-specific expression profiles of the two *FGF4* retrogenes (with associated different clinical phenotypes) may, therefore, be more dependent on the genomic context at the different insertion sites.

Dachshunds are the most commonly affected breed with IVDD, and most of the demographic and breed selection related studies have been conducted relating to the various Dachshund breed varieties [13,24,33,34]. Dachshunds, similarly, made up the largest portion of this study population, accounting for 31.6% of all retrospectively collected surgical cases, while only making up 4.7% of the DNA repository. While Dachshund variety information was not defined in this study, weights indicated that only two of the 148 surgical cases were Standard Dachshunds, while 146 were miniatures. The allele frequency for 12-*FGF4*RG was high within the breed; however, there was variation depending on where the samples were collected (0.98 in USA/UK samples and 0.94 in Swiss samples), indicating that some populations may be less homozygous than others. It is possible that some varieties of Dachshund segregate 12-*FGF4*RG more than others, and studies on IVDD in Dachshunds outside the USA suggest that this may be true: A previous genetic analysis of disc calcification in Wirehaired Standard Dachshunds registered to the Danish Kennel Club identified an associated region on chromosome 12 near the 12-*FGF4*RG, indicating that the population studied likely segregated 12-*FGF4*RG [35], and wire-haired varieties also appear to be less often clinically affected by IVDD [18,21,33,36].

Several studies have defined incidence of calcification in Dachshunds, the relationship between calcification and risk of clinical disc disease, and the heritability of disc calcification, providing a body of data to try and inform selective breeding to reduce IVDD incidence in the breed [15,21,37]. Interestingly, recent pilot data (Proschowsky and Fredholm, Gravhunden 1-2018 pp 12-13, Magazine for members of the Danish Dachshund Club) evaluating genotype of 12-*FGF4*RG and calcification scores in Dachshunds from Denmark showed an OR of 6.1 for high calcification scores (K6–K15) associated with either one or two copies of 12-*FGF4*RG allele within the wire haired variety. It is possible that data from historical calcification and heritability studies, particularly when segregating varieties were included, may have been a reflection of 12-*FGF4*RG allele frequency within the heterogeneous Dachshund populations studied [15,21,37].

Determining whether disease incidence as opposed to age of onset increases with two versus one copy of the retrogene is important information for the purpose of IVDD breed eradication strategies. Circumstantial evidence may support an increase in disease incidence with two copies given the association between historical calcification scoring (at a defined age) and disease incidence in one breed of dog [13,14], and the correlation of radiographically calcified discs and 12-*FGF4*RG allele frequency in this study. However, age at surgery in this study was highly variable, and data relating to radiographic presence of calcification should be interpreted in this context, since it has been shown that the number of radiographically calcified discs declines with age [21,38]. Selection against higher numbers of disc calcifications through radiographic screening programs in Dachshunds has been implemented in some countries as a way of reducing the incidence of IVDD [14,38], although progress has been limited to date [15]. This may reflect the inherent sensitivity and specificity issues associated with using disc calcification scoring, and its application over potentially heterogeneous 12-*FGF4*RG populations of Dachshund varieties. Among the Dachshund surgical cases in this study, 9.4% had no radiographic evidence of disc calcification. These results are similar to previous retrospective studies in Dachshunds and Pekingese that found that 13% and 17% of cases with disc extrusions had no radiographic calcification [39,40], likely reflecting low sensitivity (0.3–0.6) compared to histopathological assessment, as well as limitations of calcification as the sole marker for “clinically” relevant pathology [41,42].

## 5. Conclusions

Here, we report that 12-*FGF4*RG is both associated with intervertebral disc calcification and with age at time of surgery for IVDD across all affected breeds. The presence of 12-*FGF4*RG increases the risk for disc herniation 5.5–15.1-fold over the background risk in segregating and mixed breeds. Our findings suggest that breeding priorities should be for dogs with fewer copies of 12-*FGF4*RG, so that the allele frequency can be reduced. In breeds with lower allele frequencies of 12-*FGF4*RG, selection against the allele should reduce the incidence of disc disease. Even among breeds with high allele frequencies, genetic screening may be desirable to identify dogs with only one copy of 12-*FGF4*RG so that dogs with zero copies may eventually be bred, significantly improving the overall health of affected breeds.

## Figures and Tables

**Table 1:** Descriptive statistics for dogs surgically treated for intervertebral disc disease (IVDD). Any breed with fewer than three retrospective surgery cases was included in ’Other’. Dogs from 61 different breeds and 127 mixed breed dogs were defined. The total number of dogs from each breed in the DNA repository is included, and the list is sorted by the breed prevalence of surgical cases.

Breed	Retrospective Surgery Cases	Percent of Total Surgeries	Total in Repository	Surgery Prevalence in Repository	Prospective Surgery Cases	12-FGF4RG Frequency	18-FGF4RG Frequency	Median Age at Surgery (Years)
Dachshund	86	31.62%	221	38.91%	62	0.99	0.99	6.5
Bulldog, French	20	7.35%	81	24.69%	40	0.94	0.01	3.7
Miniature Pinscher	6	2.21%	29	20.69%	0	0.00	0.00	10.3
Pekingese	3	1.10%	17	17.65%	1	0.50	0.88	6.1
Basset Hound	5	1.84%	36	13.89%	1	0.83	1.00	5.5
Beagle	9	3.31%	65	13.85%	8	1.00	0.00	7.9
Welsh Corgi, Pembroke	6	2.21%	54	11.11%	6	0.92	1.00	7.0
Maltese	5	1.84%	65	7.69%	4	0.39	1.00	6.3
Shih Tzu	7	2.57%	92	7.61%	11	0.56	0.92	6.9
Bichon Frise	4	1.47%	59	6.78%	4	0.50	0.75	8.2
Chihuahua	9	3.31%	136	6.62%	16	0.48	0.70	6.0
Pit Bull Terrier	6	2.21%	119	5.04%	5	0.14	0.00	8.0
Cocker Spaniel, American	3	1.10%	61	4.92%	1	1.00	0.00	7.0
Doberman Pinscher	3	1.10%	70	4.29%	6	0.00	0.00	7.8
Rottweiler	4	1.47%	107	3.74%	1	0.00	0.00	5.7
Mixed Breed	46	16.91%	1316	3.50%	81	0.56	0.44	5.5
German Shepherd	5	1.84%	214	2.34%	5	0.05	0.00	6.9
Other	33	12.13%	1430	2.31%	40	0.25	0.23	7.7
Labrador Retriever	12	4.41%	568	2.11%	5	0.03	0.00	8.5
**Total**	272		4740		297	0.636	0.509	6.4

**Table 2:** Linear regression identifying characteristics associated with age at time of surgery. Standard Error (SE); Confidence Interval (CI).

	Age at IVDD Surgery		Univariable (Unadjusted) Linear Regression			Multivariable (Adjusted) Linear Regression		
	Mean	SE	Difference ^1^	95% CI	*p*	Difference ^1^	95% CI	*p*
**12-*FGF4*RG**								
zero copies	102.6	2.7	Reference			Reference		
one copy	73.5	3	−29.1	−37.1 to −21.0	<0.001	−26.6	−35.9 to −17.2	<0.001
two copies	73	2	−29.5	−36.4 to −22.7		−31.2	−40.5 to −21.8	
**Breed**								
Mixed breed	74.7	3.2	Reference	0.0 to 16.2		Reference	2.6 to 23.3	
Dachshund	82.8	2.7	8.1	−35.8 to −14.8	<0.001	12.9	−36.7 to −12.1	<0.001
French bulldog	49.4	3.1	−25.3	8.0 to 22.7		−24.4	−2.1 to 13.4	
Other purebred	90.1	2.3	15.4			5.7		
**Body weight** (5 kg)	80.4	1.5	1.6	0.3 to 2.9	0.017	−2.2	−3.8 to −0.6	0.008
**18-*FGF4*RG**								
zero copies	82.1	2.4	Reference	−22.1 to −3.2		Reference	−23.5 to −2.6	
one copy	69.4	3.9	−12.7	−6.3 to 6.3	0.02	−13.1	−14.7 to 3.7	0.049
two copies	82.1	2.2	0			−5.5		
**Sex**								
Female	78	2.4	Reference		0.158			
Male	82.3	1.9	4.3	−1.7 to 10.3				
**Reproductive Status**								
Intact	82.1	3.8	Reference		0.639			
Spayed/Neutered	80.1	1.6	−2.0	−10.4 to 6.4				

^1^ Difference column indicates the estimated difference in age (in months) at surgery compared with the reference level. Any positive value indicates later age at surgery, and any negative value indicates earlier age at surgery, versus reference level.

**Table 3:** IVDD phenotypes among surgical cases. Dogs included in Group A had evidence of calcified intervertebral discs either radiographically or at the time of surgery, while dogs categorized as Group B had no evidence of disc calcification on radiograph or at time of surgery.

Diagnosis	Count	Median Weight (kg)	Median Age at Surgery (Years)	12-*FGF4*RG Frequency	18-*FGF4*RG Frequency
Group A	378	8.1	5.5	0.765	0.587
Group B	47	25.0	9.0	0.149	0.160

**Table 4:** Logistic regression analysis of factors associated with radiographically calcified discs. Results of univariable and multivariable logistic regression analysis of factors associated with having at least one radiographically calcified disc at the time of IVDD surgery in 423 dogs. Odds Ratio (OR).

	Univariable Logistic Regression			Multivariable Logistic Regression		
	OR	95%CI	*p*	OR	95%CI	*p*
**12-*FGF4*RG**						
zero copies	Reference		<0.001	Reference		<0.001
one copy	7.75	4.04 to 14.84		6.02	2.75 to 13.18	
two copies	24.64	13.67 to 44.42		14.82	6.46 to 34.04	
**Age at IVDD**, year	0.81	0.75 to 0.87	<0.001	0.88	0.80 to 0.95	0.003
**Breed**						
Mixed breed	Reference		<0.001	Reference		0.035
Dachshund	5.8	2.70 to 12.47		1.82	0.61 to 5.39	
French Bulldog	1.46	0.69 to 3.09		0.36	0.13 to 1.02	
Other purebred	0.42	0.24 to 0.72		0.72	0.34 to 1.51	
**18-*FGF4*RG**						
zero copies	Reference			Reference		
one copy	1.88	0.94 to 0.374	<0.001	0.66	0.26 to 1.70	0.641
two copies	4.37	2.80 to 6.83		1.04	0.49 to 2.20	
**Body weight** (5 kg)	0.78	0.70 to 0.84	<0.001	-	-	-
**Male sex**	0.99	0.67 to 1.47	0.96	-	-	-
**Spayed or neutered**	2.1	1.23 to 3.56	0.006	-	-	-

**Table 5:** Logistic regression analysis of factors associated with IVDD surgery in 506 mixed breed dogs (46 surgical cases and 460 non-surgical). All non-surgical cases were at least 10 years of age at their last visit to the Veterinary Medical Teaching Hospital (VMTH) and had no diagnosis of IVDD.

	Univariable Logistic Regression			Multivariable Logistic Regression		
	OR	95%CI	*p*	OR	95%CI	*p*
**12-*FGF4*RG**						
zero copies	Reference	-	<0.001	Reference	-	<0.001
one copy	18.38	8.51 to 43.26		18.42	7.44 to 50.26	
two copies	43.11	15.10 to 129.8		44.23	12.92 to 163.3	
**18-*FGF4*RG**						
zero copies	Reference	-	<0.001	Reference	-	0.079
one copy	4.9	2.36 to 10.19		3.05	1.14 to 8.34	
two copies	7.7	3.25 to 17.46		2.3	0.77 to 6.90	
**Body weight** (5 kg)	0.7	0.59 to 0.81	<0.001	1.15	0.92 to 1.44	0.22
**Male sex**	1.33	0.73 to 2.48	0.355	-	-	-

**Table 6:** Relative risk associated with 12-*FGF4*RG for IVDD. Dogs included in the IVDD categories were at least 10 years of age as of their last visit to the VMTH. Breed allele frequencies information is taken from dogs with no diagnosis of IVDD (Supplementary Table S1).

Breed	Total Dogs	12-*FGF4*RG Allele Frequency	Relative Risk	95% CI	*p* Value
Shih Tzu	52	0.25	10.3	1.8–62.1	0.005
Bichon Frise	39	0.18	10.0	1.7–60.1	0.011
Mixed breed	508	0.10	15.1	7.6–29.9	<0.0001
Chihuahua	60	0.10	5.5	1.7–18.2	0.008

## Supplementary Materials

The following are available online at https://www.mdpi.com/2073-4425/10/6/435/s1, Figure S1: Anatomical localization of surgical procedures in dogs, Table S1: Breed list with genotypes for 12-*FGF4*RG and 18-*FGF4*RG.
