# Supplementary material for: Phenotypic Effects of FGF4 Retrogenes on Intervertebral Disc Disease in Dogs
Source: Genes (Basel). 2019 Jun 7;10(6):435. doi: 10.3390/genes10060435 (PMC6627552; doi:10.3390/genes10060435)
Supplement: Supplementary file 1 [file genes-10-00435-s001.zip › Table_S1.docx]

|  |  | **12-*FGF4*RG** | | | | **18-*FGF4*RG** | | | |
| --- | --- | --- | --- | --- | --- | --- | --- | --- | --- |
| Breed | **Total** | **0** | **1** | **2** | **Frequency** | **0** | **1** | **2** | **Frequency** |
| Alaskan Malamute | 21 | 21 | 0 | 0 | 0 | 21 | 0 | 0 | 0 |
| Alpine Dachsbracke | 174 | 25 | 93 | 56 | 0.59 | 1 | 112 | 61 | 0.67 |
| Appenzeller Sennenhund | 121 | 121 | 0 | 0 | 0 | 121 | 0 | 0 | 0 |
| Australian Cattle Dog | 11 | 11 | 0 | 0 | 0 | 5 | 0 | 0 | 0 |
| Australian Shepherd | 46 | 44 | 2 | 0 | 0.02 | 37 | 0 | 0 | 0 |
| Basset Griffon Vendéen Petit | 10 | 10 | 0 | 0 | 0 | 0 | 0 | 10 | 1.00 |
| Basset Hound | 32 | 3 | 18 | 11 | 0.63 | 1 | 4 | 27 | 0.91 |
| Bavarian Mountain Hound | 69 | 1 | 10 | 58 | 0.91 | 68 | 0 | 0 | 0 |
| Beagle | 12 | 0 | 0 | 12 | 1.00 | 5 | 0 | 0 | 0 |
| Bernese Mountain Dog | 11 | 11 | 0 | 0 | 0 | 5 | 0 | 0 | 0 |
| Bichon Frise | 71 | 49 | 19 | 3 | 0.18 | 1 | 2 | 5 | 0.75 |
| Border Collie | 5 | 5 | 0 | 0 | 0 | 5 | 0 | 0 | 0 |
| Border Terrier | 6 | 6 | 0 | 0 | 0 | 6 | 0 | 0 | 0 |
| Boston Terrier | 5 | 5 | 0 | 0 | 0 | 5 | 0 | 0 | 0 |
| Brittany | 17 | 17 | 0 | 0 | 0 | 6 | 0 | 0 | 0 |
| Bull Terrier | 5 | 5 | 0 | 0 | 0 | 5 | 0 | 0 | 0 |
| Bulldog, English | 13 | 13 | 0 | 0 | 0 | 5 | 0 | 0 | 0 |
| Bulldog, French | 106 | 0 | 7 | 99 | 0.97 | 12 | 1 | 0 | 0.04 |
| Cairn Terrier | 10 | 10 | 0 | 0 | 0 | 0 | 1 | 9 | 0.95 |
| Cavalier King Charles Spaniel | 21 | 0 | 0 | 21 | 1.00 | 5 | 0 | 0 | 0 |
| Chesapeake Bay Retriever | 40 | 34 | 6 | 0 | 0.08 | 9 | 0 | 0 | 0 |
| Chihuahua | 199 | 163 | 34 | 2 | 0.10 | 2 | 4 | 31 | 0.89 |
| Chinese Crested | 9 | 5 | 4 | 0 | 0.22 | 8 | 1 | 0 | 0.06 |
| Clumber Spaniel | 5 | 0 | 0 | 5 | 1.00 | 5 | 0 | 0 | 0 |
| Cocker Spaniel, American | 9 | 0 | 1 | 8 | 0.94 | 5 | 0 | 0 | 0 |
| Cocker Spaniel, English | 14 | 0 | 1 | 13 | 0.96 | 14 | 0 | 0 | 0 |
| Coton de Tulear | 12 | 2 | 6 | 4 | 0.58 | 0 | 1 | 11 | 0.96 |
| Dachshund (Swiss) | 136 | 0 | 16 | 120 | 0.94 | 0 | 5 | 131 | 0.98 |
| Dachshund (US/UK) | 257 | 0 | 10 | 247 | 0.98 | 1 | 2 | 240 | 0.99 |
| Dandie Dinmont Terrier | 27 | 1 | 4 | 22 | 0.89 | 0 | 0 | 27 | 1.00 |
| Danish Swedish Farmdog | 29 | 23 | 6 | 0 | 0.10 | 12 | 0 | 0 | 0 |
| Doberman Pinscher | 14 | 14 | 0 | 0 | 0 | 5 | 0 | 0 | 0 |
| Entlebucher Mountain Dog | 8 | 0 | 5 | 3 | 0.69 | 8 | 0 | 0 | 0 |
| Fox Terrier | 11 | 11 | 0 | 0 | 0 | 6 | 0 | 1 | 0.14 |
| German Hound | 16 | 1 | 9 | 6 | 0.66 | 16 | 0 | 0 | 0 |
| German Shepherd Dog | 15 | 15 | 0 | 0 | 0 | 5 | 0 | 0 | 0 |
| Glen of Imaal Terrier | 8 | 8 | 0 | 0 | 0 | 0 | 0 | 8 | 1.00 |
| Golden Retriever | 14 | 14 | 0 | 0 | 0 | 6 | 0 | 0 | 0 |
| Great Dane | 12 | 12 | 0 | 0 | 0 | 5 | 0 | 0 | 0 |
| Irish Setter | 8 | 8 | 0 | 0 | 0 | 5 | 0 | 0 | 0 |
| Jack Russel Terrier | 12 | 10 | 2 | 0 | 0.08 | 3 | 0 | 0 | 0 |
| Labrador Retriever | 21 | 21 | 0 | 0 | 0 | 11 | 0 | 0 | 0 |
| Lagotto Romagnolo | 53 | 53 | 0 | 0 | 0 | 53 | 0 | 0 | 0 |
| Maltese | 87 | 83 | 3 | 1 | 0.03 | 0 | 0 | 18 | 1.00 |
| Mixed Breed | 572 | 474 | 81 | 17 | 0.10 | 466 | 73 | 33 | 0.12 |
| Newfoundland | 13 | 13 | 0 | 0 | 0 | 5 | 0 | 0 | 0 |
| Norwich Terrier | 19 | 19 | 0 | 0 | 0 | 3 | 0 | 16 | 0.84 |
| Nova Scotia Duck Tolling Retriever | 172 | 69 | 87 | 16 | 0.35 | 7 | 0 | 0 | 0 |
| Pekingese | 28 | 5 | 11 | 12 | 0.63 | 1 | 0 | 19 | 0.95 |
| Pinscher, Miniature | 9 | 8 | 1 | 0 | 0.06 | 9 | 0 | 0 | 0 |
| Podengo Pequeno | 7 | 7 | 0 | 0 | 0 | 0 | 1 | 6 | 0.93 |
| Poodle, Miniature and Toy | 114 | 28 | 43 | 43 | 0.57 | 37 | 7 | 4 | 0.16 |
| Poodle, Standard | 55 | 55 | 0 | 0 | 0 | 30 | 0 | 1 | 0.03 |
| Portuguese Water Dog | 12 | 9 | 3 | 0 | 0.13 | 5 | 0 | 0 | 0 |
| Pug | 7 | 7 | 0 | 0 | 0 | 7 | 0 | 0 | 0 |
| Rottweiler | 10 | 10 | 0 | 0 | 0 | 5 | 0 | 0 | 0 |
| Russell Terrier | 10 | 10 | 0 | 0 | 0 | 0 | 4 | 6 | 0.80 |
| Russian Tsvetnaya Bolonka | 6 | 4 | 2 | 0 | 0.17 | 0 | 2 | 4 | 0.83 |
| Schnauzer, Miniature | 8 | 8 | 0 | 0 | 0 | 8 | 0 | 0 | 0 |
| Schweizer Laufhund | 65 | 51 | 14 | 0 | 0.11 | 65 | 0 | 0 | 0 |
| Schweizerischer Niederlaufhund | 46 | 12 | 21 | 13 | 0.51 | 16 | 17 | 13 | 0.47 |
| Scottish Terrier | 12 | 11 | 1 | 0 | 0.04 | 0 | 1 | 6 | 0.93 |
| Shetland Sheepdog | 13 | 13 | 0 | 0 | 0 | 5 | 0 | 0 | 0 |
| Shih Tzu | 110 | 64 | 36 | 10 | 0.25 | 0 | 0 | 5 | 1.00 |
| Siberian Husky | 11 | 11 | 0 | 0 | 0 | 5 | 0 | 0 | 0 |
| Skye Terrier | 13 | 2 | 2 | 9 | 0.77 | 0 | 0 | 9 | 1.00 |
| Springer Spaniel, English | 22 | 9 | 8 | 5 | 0.41 | 12 | 0 | 0 | 0 |
| St. Bernard | 10 | 10 | 0 | 0 | 0 | 5 | 0 | 0 | 0 |
| Weimaraner | 13 | 13 | 0 | 0 | 0 | 5 | 0 | 0 | 0 |
| Welsh Corgi, Cardigan | 6 | 1 | 1 | 4 | 0.75 | 0 | 1 | 4 | 0.90 |
| Welsh Corgi, Pembroke | 51 | 3 | 13 | 35 | 0.81 | 0 | 2 | 49 | 0.98 |
| West Highland White Terrier | 10 | 10 | 0 | 0 | 0 | 0 | 0 | 8 | 1.00 |
| Whippet | 5 | 5 | 0 | 0 | 0 | 5 | 0 | 0 | 0 |
| Yorkshire Terrier | 12 | 11 | 1 | 0 | 0.04 | 0 | 0 | 5 | 1.00 |
| TOTAL | 3223 | 1787 | 581 | 855 | 0.36 | 1175 | 240 | 766 | 0.41 |
