# Supplementary figures and images for: Phenotypic Effects of FGF4 Retrogenes on Intervertebral Disc Disease in Dogs
Source: Genes (Basel). 2019 Jun 7;10(6):435. doi: 10.3390/genes10060435 (PMC6627552; doi:10.3390/genes10060435)

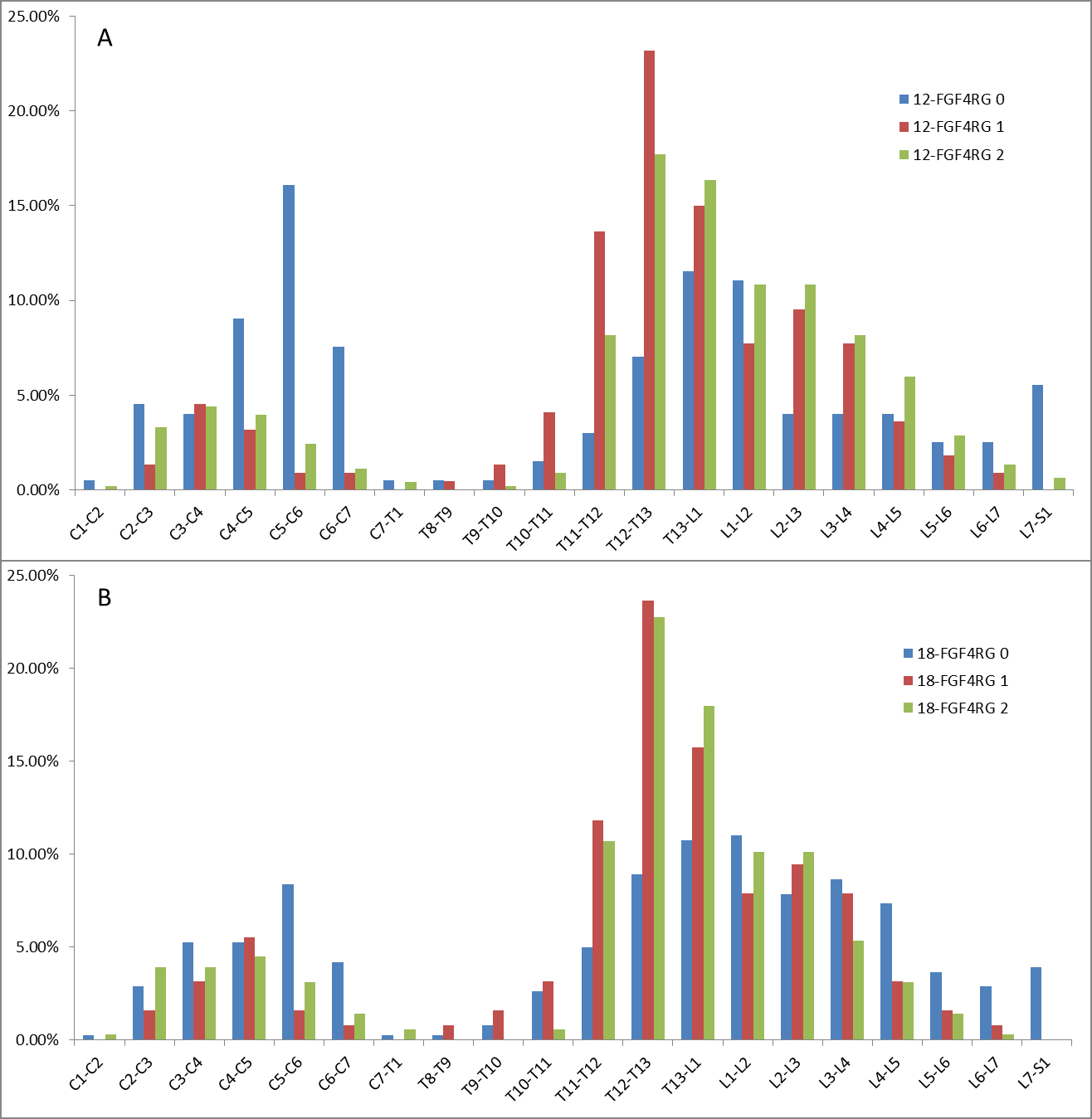

Supplement: Supplementary file 1 [file genes-10-00435-s001.zip › Figure_S1.tif]
